# Fibrinogen Adsorption and Sponge-like Aggregate Formation on Titanium Modified by Electrochemically Deposited CaCO_3_

**DOI:** 10.3390/jfb17080366

**Published:** 2026-07-30

**Authors:** Zubair Ahmed, Huiliang Cao

**Affiliations:** 1 Interfacial Electrochemistry and Biomaterials, School of Materials Science and Engineering, East China University of Science & Technology, Shanghai 200237, China; zubair135@mail.ecust.edu.cn; 2 Engineering Research Center for Biomedical Materials of the Ministry of Education, East China University of Science & Technology, Shanghai 200237, China; 3 Key Laboratory for Ultrafine Materials of Ministry of Education, East China University of Science & Technology, Shanghai 200237, China

**Keywords:** surface modification, electrochemical deposition, fibrin, calcium carbonate, protein adsorption

## Abstract

Fibrinogen adsorption governs biological responses to implantable medical devices; however, surface properties influence the overall functionality of the biomaterial, and guided fibrinogen adsorption remains limited. In the present work, CaCO_3_ was electrochemically deposited on commercial Ti at −1.6 V for 1 h, 2 h, and 3 h. Furthermore, the effects on fibrinogen adsorption were detailed by using dye-assisted scanning electron microscopy (*d*-SEM), X-ray photoelectron spectroscopy (*XPS*), and Fourier Transform Infrared Spectroscopy (*ATR-FTIR*). Longer deposition times produced thicker calcite layers with maximum surface coverage of 99.80 ± 0.40%, accompanied by progressively greater calcium-ion release, ranging from 3.5 mg·L^−1^·cm^−2^ (1 h) to 12.2 mg·L^−1^·cm^−2^ (3 h) over 240 min. The results show that electrochemically deposited calcite crystals for 3 h lead to the formation of sponge-like fibrinogen aggregates via calcium ion-mediated conformational activation, particularly by chelating with the histidine and carboxylate residues of the Bβ chain segment Gly-His-Arg-Pro (β15–β18). This structural reorganization was supported by XPS N 1s binding energy at 398.80 eV and a red shift in Amide I and Amide II bands in FTIR spectra. Overall, this study reveals that careful modification of surface chemistry can guide fibrinogen adsorption, which can be beneficial for advanced biomaterials to orchestrate tissue integration at the protein and cellular levels.

## 1. Introduction

Over the past few decades, surface modification technologies have advanced rapidly and have become a major contributor to biomaterial development. The successful application of these technologies depends on a fundamental understanding of the interactions between biomolecules and biomaterial surfaces [1,2,3]. Fibrinogen, a key plasma protein in the body’s hemostatic system, is synthesized by hepatocytes and circulates in plasma at a concentration of approximately 2~4 mg/mL [4]. Fibrinogen adsorption onto biomaterial surfaces plays a pivotal role in directing the earliest cellular and biological reactions at the material–tissue boundary. Once a biomaterial is implanted, its surface is rapidly exposed to physiological fluids, where proteins, including fibrinogen, adsorb within seconds, forming the initial biointerface. This adsorbed protein layer governs biocompatibility, inflammatory signaling, thrombosis, and ultimately clinical performance [5]. These fibrinogen roles are mediated by specific binding motifs that are frequently hidden within the native protein structure but become exposed upon adsorption to material surfaces. Consequently, controlling fibrinogen adsorption is a hot topic in the development of advanced biomaterials. It is reported that the kinetics of fibrinogen adsorption on pristine biomaterials depend on surface chemistry [6], crystal orientation [7], nanoscale roughness [8], and surface charge [9], and these factors can induce conformational rearrangement of the adsorbed fibrinogen. Such conformational rearrangements are influenced not only by intrinsic surface properties but also by electrostatic interactions, local ionic conditions, and the resulting protein surface charge interactions [10]. For example, our previous study demonstrated that calcium doping of titanium modulates the conformation of adsorbed fibrinogen, thereby exposing the protein’s antibacterial peptide motifs that inhibit *Pseudomonas aeruginosa* colonization [11]. Nevertheless, established strategies for directing and controlling fibrinogen adsorption on solid surfaces are limited. Since fibrinogen adsorbs immediately as an intrinsic part of material–tissue contact, developing methods to control its adsorption on implantable materials and to harness this natural process is critically important.

Titanium is widely used in medical applications because of its low density, high strength, and excellent biocompatibility. Various surface modification approaches, including physical (ion deposition), mechanical (polishing), and chemical (acid etching) procedures, are employed to tailor implant surface properties and regulate their interactions with cells and tissues [12]. Within the broader category of chemical modification, electrochemical deposition is a particularly versatile approach because it is driven by interfacial electrochemical reactions that promote localized nucleation and growth directly on the titanium substrate [13]. Compared with sol–gel and hydrothermal methods, it offers a low-temperature, time-efficient, low-cost, and scalable route to produce uniform, adherent, and conformal coatings on complex implant geometries, while also enabling precise control over coating thickness and microstructure through adjustment of deposition parameters (i.e., temperature, pH, and deposition time) [14]. Unlike physical or mechanical surface treatments that mainly modify topography, electrochemical deposition combines control over ion transport with morphological tunability, thereby ensuring strong interfacial adhesion and reproducible coating formation. This controlled interfacial chemistry provides a platform for regulating surface properties in a manner directly relevant to biological interactions at the implant interface [13]. In this context, coating titanium with the bioactive material, such as calcium carbonate (CaCO_3_), has emerged as a promising strategy to modify surface characteristics and biological responses [15]. Calcium carbonate (CaCO_3_) has garnered substantial interest in both academic research and industrial applications due to its structural versatility, tunable morphologies, diverse functionalities, pH responsiveness, and inherent biocompatibility [16]. It exists in three crystalline polymorphs, calcite, aragonite, and vaterite, each with distinct properties. Vaterite offers high surface area and solubility with rapid Ca^2+^ release, aiding cell signaling and repair. Aragonite, structurally akin to bone mineral, enhances osteoblast activity. Calcite is widely applied in drug delivery and bone scaffolds [17]. For example, Victoriya et al. synthesized CaCO_3_ nanoparticles (≤200 nm) and evaluated their interaction with doxorubicin (DOX). The study demonstrated sustained DOX release from the CaCO_3_ nanocarriers, effectively inhibiting cancer cell growth in vitro [18]. In another study by Li Thi et al., CaCO_3_ coatings fabricated on titanium substrates demonstrated enhanced osteoblast cell responses, proliferation, and bone-like nodule formation compared to uncoated titanium substrates [19]. Recently, Ana et al. developed a micro-sized hybrid of calcium carbonate and Ag nanoparticles and demonstrated antibacterial activity against Escherichia coli, methicillin-resistant Staphylococcus aureus (MRSA), and Pseudomonas aeruginosa, three bacteria responsible for many healthcare infections, without affecting human fibroblast and stem cell viability [20]. Very recently, Peng et al. demonstrated that fibrinogen-encapsulated CaCO_3_ particles exhibit superior hemostatic activities when combined with tranexamic acid [21]. These findings position CaCO_3_ not merely as a bioactive coating but as a multifunctional platform whose protein interactions and biological signaling can be engineered through polymorph control and deposition methods. Although previous studies have demonstrated the bioactivity, antibacterial performance, drug-loading capability, and hemostatic potential of CaCO_3_-based materials, they have not clarified the role of electrochemically deposited CaCO_3_ coatings on titanium in regulating fibrinogen adsorption and interfacial assembly. In particular, the effects of coating deposition time on surface chemistry, Ca-mediated protein coordination, and fibrinogen conformational behavior remain unresolved.

Accordingly, in this study, CaCO_3_ was electrochemically deposited on polished medical Ti at −1.6 V for different time intervals, and the resulting fibrinogen adsorption behavior was investigated using dye-assisted scanning electron microscopy (*d*-SEM), X-ray photoelectron spectroscopy (*XPS*), and Fourier Transform Infrared Spectroscopy (FTIR).

## 2. Materials and Methods

### 2.1. Working Electrode Preparation

To improve material properties and surface quality, commercial pure titanium (Ti) (Baoji Titanium Industry Co., Ltd.; Baoji, Shaanxi, China) was cut into rectangles (20 × 10 mm on each side and 1 mm thick) and ground using silicon carbide (SiC) polishing paper (Zibo Riken MT Coated Abrasives Co., Ltd.; Zibo, Shandong, China) until a fine grade of 900, which successfully eliminated surface irregularities and imperfections. The titanium substrates were then fine-polished with diamond paste (first with a 2 μm diamond particle size and then with a finer 0.5 μm paste) (Changzhou Huayida Tools Co., Ltd., Changzhou, Jiangsu, China). This two-step polishing process ensured a high degree of surface smoothness and added to the material’s overall luster. Metallographic cloths (Metallographic polishing cloth, Trojan (Suzhou) Material Technology Co., Ltd., Suzhou, Jiangsu, China) were used to achieve a final mirror-like finish, which improved the titanium specimens’ surface quality. After that, the polished substrates were cleaned with ethanol for 10 min in a sonicator (Shanghai Kuncheng Scientific Instrument Co., Ltd., Shanghai, China), washed with distilled water for 10 min in a sonicator, and air-dried at room temperature before electrochemical deposition [22].

### 2.2. Electrolyte Preparation

Calcium chloride (CaCl_2_), sodium bicarbonate (NaHCO_3_), and sodium chloride (NaCl) are analytical reagents used directly (Sigma-Aldrich, Trading Co., Ltd.; Shanghai, China). Deionized water was used throughout the experiments. The electrolyte solution was composed of 0.2 M CaCl_2_, 0.2 M NaHCO_3_, and 1.0 M NaCl, designed to replicate ionic conditions relevant to biomineralization. The pH was imposed at 8.25 by careful addition of 0.1 M NaOH (Shanghai Aladdin Biochemical Technology Co., Ltd., Shanghai, China) thereby maintaining carbonate stability and ensuring reproducible electrochemical deposition of CaCO_3_ coatings on titanium substrates.

### 2.3. Procedures and Apparatus for CaCO_3_ Deposition

A conventional three-electrode system at 26 °C was adopted for the electrochemical deposition. Cp Ti and a graphite sheet (three rectangular sheets with fixed dimensions of 3 cm (width) × 4 cm (length)) acted as the working and counter electrodes, respectively, while Ag/AgCl was used as the reference electrode. The linear sweep voltammetry (LSV) test was performed (a scan rate of 2.0 mV/s on a Shanghai Chenhua Instrument Co., Ltd., Shanghai, China) to determine the optimal deposition potential. Thus, CaCO_3_ coating was produced by applying a constant negative potential of −1.6 V to the Ti working electrode at 26 °C for 1 h (designated as TiCaCO_3_/1h), 2 h (designated as TiCaCO_3_/2h), and 3 h (designated as TiCaCO_3_/3h).

### 2.4. Materials Characterization

***Calcium release analysis*:** Samples with an exposed surface area of 1 cm^2^ were immersed in 10 mL of calcium-free PBS and incubated at 37 °C for 30, 60, 120, and 240 min. At each time point, the incubation medium was replaced, and the collected aliquots were retained for calcium concentration determination by inductively coupled plasma optical emission spectrometry (ICP-OES) using an Agilent 5800 VDV (Agilent, Johar, Malaysia) system, which has a 0.01 ppm calcium detection limit. Calibration curves were generated using certified Ca^2+^ reference standards to ensure traceable and accurate quantification. Before analysis, all collected solutions were filtered using 0.2 μm filters to remove any particulate matter.

***Surface morphology*:** A scanning electron microscope (SEM; HITACHI, S-4800, Tokyo, Japan) operating at an accelerating voltage of 5 kV and a working distance of 5 mm was used to investigate the surface morphology of the materials.

***X-ray diffraction (XRD)*:** X-ray diffraction (XRD) measurements were carried out using a D8 Advance ECO diffractometer (Bruker, Beijing Technology Co., Ltd., Beijing, China), operated at 40 kV and 25 mA. A 2θ scan from 5° to 80° was performed.

***X-ray photoelectron spectroscopy (XPS)-based surface composition analysis*:** The composition and structural changes in fibrinogen adsorbed on electrochemically deposited CaCO_3_ coatings on Ti samples prepared for different time intervals were characterized by X-ray photoelectron spectroscopy (XPS; Nexsa, Thermo Fisher Scientific, Waltham, Massachusetts, USA) using a monochromatic Al Kα source (1486.6 eV) operated at 150 W.

***Fourier Transform Infrared Spectroscopy (ATR-FTIR)*:** Fourier-transform infrared (FTIR) spectra were recorded in the 4000~400 cm^−1^ range using an attenuated total reflection (ATR) accessory attached to a Nicolet iS50 spectrometer (Thermo Scientific, Waltham, MA, USA). Measurements were performed at a spectral resolution of 8 cm^−1^. The spectral regions containing relevant information were deconvoluted using a Gaussian band-fitting profile in Origin 2022 software.

***Statistical Analysis*:** Origin was used for all statistical evaluations. For crystal size, *n* = 30 measurements were analyzed, and for surface coverage, *n* = 10 independent replicates were included, and for thickness, *n* = 20 independent replicates were included. A one-way ANOVA followed by Tukey’s post hoc test was applied to compare deposition times, and statistical significance was defined as *p* < 0.05.

### 2.5. Fibrinogen Adsorption Assays

***Quantification of fibrinogen stock solutions*:** A fibrinogen stock suspension was prepared by dissolving 4 mg of freeze-dried human plasma fibrinogen (Millipore 341578, Sigma-Aldrich, Shanghai, China) in 4 mL of calcium-free phosphate-buffered saline (PBS, BioSharp, Beijing, China) in a centrifuge tube at room temperature until fully dissolved. For ultraviolet (UV) spectrophotometric analysis, 0.5 mL of the stock suspension was diluted to 5 mL with PBS to obtain a 10-fold dilution, and the UV absorbance at 280 nm was recorded using a UV spectrophotometer (Shanghai Xipu Instrument Co., Ltd., Shanghai, China) with a 1 cm quartz cuvette. At this wavelength, the tryptophan and tyrosine residues in proteins show strong absorbance, allowing the fibrinogen concentration to be quantified using the Lambert–Beer law (Equation (1)).

(1) A=ε⋅c⋅L

Here, **(*A*)** is the absorbance at 280 nm, **(*c*)** is the fibrinogen concentration, **(*L*)** is the cuvette path length in centimeters, and ***ε*** is the extinction coefficient at 280 nm, which equals 1.51 mL per mg per cm for human plasma fibrinogen [23]. Using these values, the fibrinogen concentration of the stock suspension was determined. The stock was then diluted with PBS for the adsorption and probe assays. Working fibrinogen concentrations in this study were 1 mg·L^−1^ (1Fib), 5 mg·L^−1^ (5Fib), and 10 mg·L^−1^ (10Fib).

***Dye-assisted fibrinogen incubation*:** Then, samples were incubated (Shanghai Kuncheng Scientific Instrument Co., Ltd., Shanghai, China) at 37 °C for 1 h in 5 mL of either PBS or dilute fibrinogen suspensions at concentrations of 1 mg·L^−1^ (1Fib), 5 mg·L^−1^ (5Fib), and 10 mg·L^−1^ (10Fib^+^). Following incubation, the samples were sequentially rinsed with PBS and ultrapure water, then subjected to negative staining with 1 mL of 1% phosphotungstic acid (PTA) for 10 min. Subsequently, all samples were rinsed twice with ultrapure water and air-dried. The surface morphologies of fibrinogen assemblies formed on the materials were examined using scanning electron microscopy.

## 3. Results

### 3.1. Linear Sweep Voltammetry for Optimal CaCO_3_ Deposition Potential

**Figure 1:**
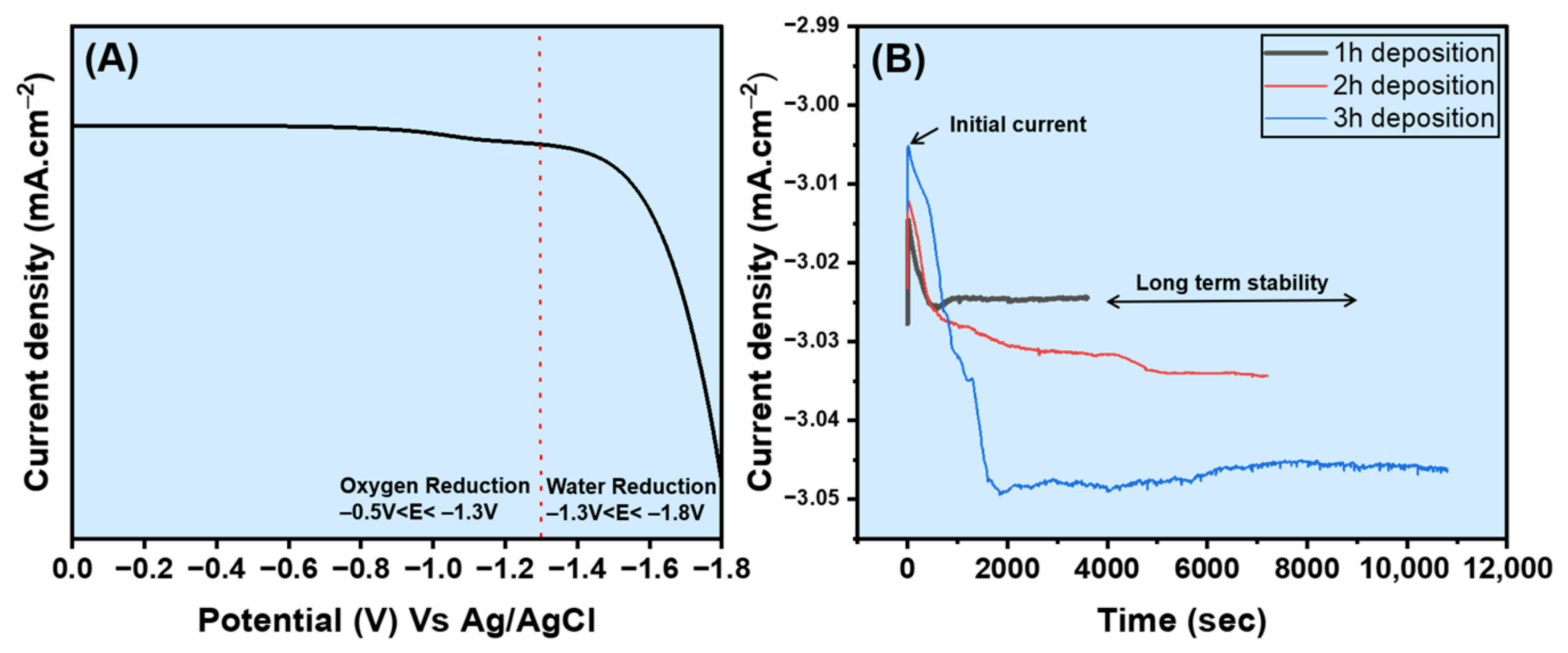
(**A**) Linear sweep voltammetry of a Ti substrate in 0.2 M CaCl_2_, 0.2 M NaHCO_3_, and 1.0 M NaCl aqueous solution, with the pH adjusted to 8.25 using 0.1 M NaOH (scan rate: 2 mV s^−1^). The arrow denotes the potential scan direction. (**B**) Amperometric *i–t* response of the Ti substrate in the same electrolyte under an applied potential of −1.6 V vs. Ag/AgCl for 1 h, 2 h, and 3 h.

Linear sweep voltammetry was performed to analyze cathodic reactions on a titanium (Ti) substrate in an electrolyte solution and confirm the optimal potential for electrodeposition. A potential sweep from 0.0 to −1.8 V vs. Ag/AgCl was applied to Ti at a scan rate of 2.0 mV s^−1^. As shown in Figure 1A, the cathodic current exhibits a diffusional plateau in the potential range of approximately −0.5 to −1.3 V vs. Ag/AgCl, which is consistent with oxygen reduction under mass-transport-limited conditions [24]. This plateau-like behavior suggests that the reduction in dissolved oxygen is the dominant cathodic process in this region; the reaction rate is limited by the low concentration and slow diffusion of oxygen in the electrolyte, as described in Equation (2).

O_2_ + 2H_2_O + 4e^−^ → 4OH^−^ (2)

When the potential is more negative than −1.3 V (vs. Ag/AgCl), the cathodic current density increases rapidly, indicating the onset of water reduction in accordance with literature [24]. At these more negative potentials, the reduction in dissolved oxygen is no longer the main cathodic process, and the abundant water molecules in the electrolyte become the dominant reducible species. Therefore, water reduction proceeds according to Equation (3).

2H_2_O + 2e^−^ → H_2_ + 2OH^−^ (3)

In this process, the OH^−^ ions are concentrated around the Ti surface, which induces an increase in the local pH value. The generated OH^−^ reacts with the HCO_3_^−^, and then the CaCO_3_ deposits are formed on the surface of the electrode according to Equations (4) and (5) [25]. To increase the surface coverage of the CaCO_3_ coatings, a relatively high negative potential (−1.6 V vs. Ag/AgCl) was used in the present study.

HCO_3_^−^ + OH^−^ → CO_3_^2−^ + H_2_O (4)

CO_3_^2−^ + Ca^2+^ → CaCO_3_ (s) (5)

### 3.2. Amperometric i-t Curves for CaCO_3_ Deposition

Amperometry is a conventional approach for analyzing the CaCO_3_ scale deposition behavior. In the amperometry test, the potential of −1.6 V (vs. Ag/AgCl) was applied to the polished Ti substrate at 26 °C. Based on the LSV result (Figure 1A), which showed that oxygen reduction dominates in the plateau region (approximately −0.5 to −1.3 V), whereas water reduction begins at potentials more negative than about −1.3 V. Since CaCO_3_ deposition is driven by cathodically generated OH^−^ and the resulting local pH increase, −1.6 V was selected to provide sufficient driving force for water reduction and thus promote efficient CaCO_3_ nucleation and growth on the Ti substrate [26]. Figure 1B shows the amperometry curves obtained for applied potentials of −1.6 V on polished Ti at 26 °C for 1 h, 2 h, and 3 h. A pronounced increase in current density was observed at the initial stage of the experiment, followed by a gradual decline with time, consistent with the transient behavior reported in previous studies [27]. The initial increase in current density is associated with the nucleation stage. During this stage, the formation of new CaCO_3_ nuclei and crystalline phases increases the electrochemically active surface area for interfacial charge transfer, increasing initial current density [28]. In addition, cathodically generated OH^−^ increases the local pH near the Ti surface, promoting carbonate formation and CaCO_3_ nucleation; therefore, the current transient reflects the coupled evolution of electrochemical reduction and deposit formation [29]. The behavior of these transients represents a typical nucleation process with three-dimensional growth of nuclei limited by diffusion of the active species [28,30]. The declining trend represents the growth of CaCO_3_ scales on the Ti surface [28]. The 1 h and 2 h deposition curves exhibit a rapid decrease in current density within the first 500 s, which is attributed to the swift nucleation and growth of CaCO_3_ crystallites that quickly block the available active sites on the Ti surface. This behavior indicates that shorter deposition runs lead to rapid surface coverage and early restriction of charge transfer. In contrast, the 3 h deposition curve decays more gradually, extending over ~2000 s, suggesting a more sustained nucleation and growth stage. The prolonged decline reflects that the electrode surface is already partially covered by pre-deposited CaCO_3_, resulting in slower nucleation kinetics and enhanced diffusion limitations. Only after sufficient film thickening does a compact CaCO_3_ layer form, which restricts both charge transfer and mass transport. Thus, although the applied potential is constant at −1.6 V, the deposition time critically governs the transition from nucleation-dominated to diffusion-controlled growth regions [31]. The TiCaCO_3_/1h coating exhibited the lowest initial current, which rapidly decayed and stabilized at a minimal baseline, indicating limited nucleation activity but greater long-term stability. The TiCaCO_3_/2h coating exhibited intermediate current values with a more sustained decay profile, suggesting balanced deposition kinetics and moderate stability. In contrast, the TiCaCO_3_/3h coating displays the highest initial and sustained current densities throughout the measurement period, consistent with enhanced nucleation, continuous ion consumption, and thicker CaCO_3_ growth.

### 3.3. Surface Characteristics of the Material

**Figure 2:**
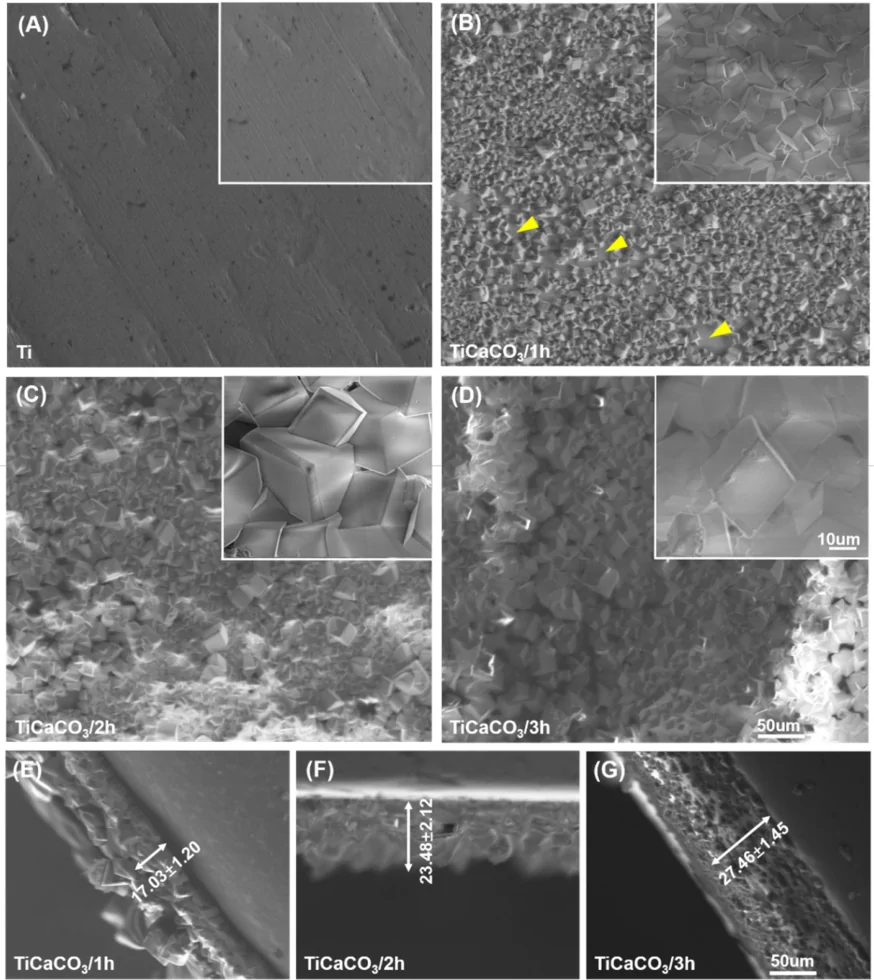
SEM morphologies of CaCO_3_ deposited at −1.6 V on Ti for different times: (**A**) Ti, (**B**) for 1 h, (**C**) for 2 h, and (**D**) for 3 h. Thickness of the CaCO_3_ layer deposited (**E**) for 1 h, (**F**) for 2 h, and (**G**) for 3 h.

To examine the morphology of the CaCO_3_ deposits on the Ti working electrode, SEM imaging was carried out. The surface of Ti was found to be flat and smooth (Figure 2A), while after CaCO_3_ deposition on all three groups, TiCaCO_3_/1h, TiCaCO_3_/2h, and TiCaCO_3_/3h deposited at −1.6 V, cubic crystals of CaCO_3_ were found (Figure 2B–D). The SEM analysis is consistent with the amperometric *i–t* curve behavior and clearly shows that deposition time controls surface coverage of the CaCO_3_ layer. At −1.6 V on TiCaCO_3_/1h, well-defined cubic crystals are present (Figure 2B inset). These crystals are relatively isolated in some places with an average size of 2.06 ± 0.48 μm (Figure S1A, Supplementary Materials), and appreciable regions of bare Ti (arrows in Figure 2B) are exposed at some points. The surface coverage by these was found to be 94.06 ± 4.45 μm (Figure S1B, Supplementary Materials). With an increase in the deposition time from 1 h to 2 h, the surface coverage by CaCO_3_ on TiCaCO_3_/2h has increased noticeably, and no bare Ti surface is visible (Figure 2C). The average crystal size is 6.75 ± 1.12 μm, and an average surface coverage of 99.73 ± 0.43% is achieved (Figure S1A,B, Supplementary Materials). When the deposition time is increased to 3 h, maximum surface coverage (99.80 ± 0.40%) (Figure S1B, Supplementary Materials) is achieved, without any phase transition on TiCaCO_3_/3h (Figure 2D), and the average crystal size is increased to 7.42 ± 2.28 μm (Figure S1A, Supplementary Materials). Moreover, the layer thickness has increased with increasing deposition time (Figure 2E–G). For 1 h, 2 h, and 3 h depositions, the coating thickness is 17.03 ± 1.20 μm, 23.48 ± 2.12 μm, and 27.46 ± 1.45 μm, respectively.

**Figure 3:**
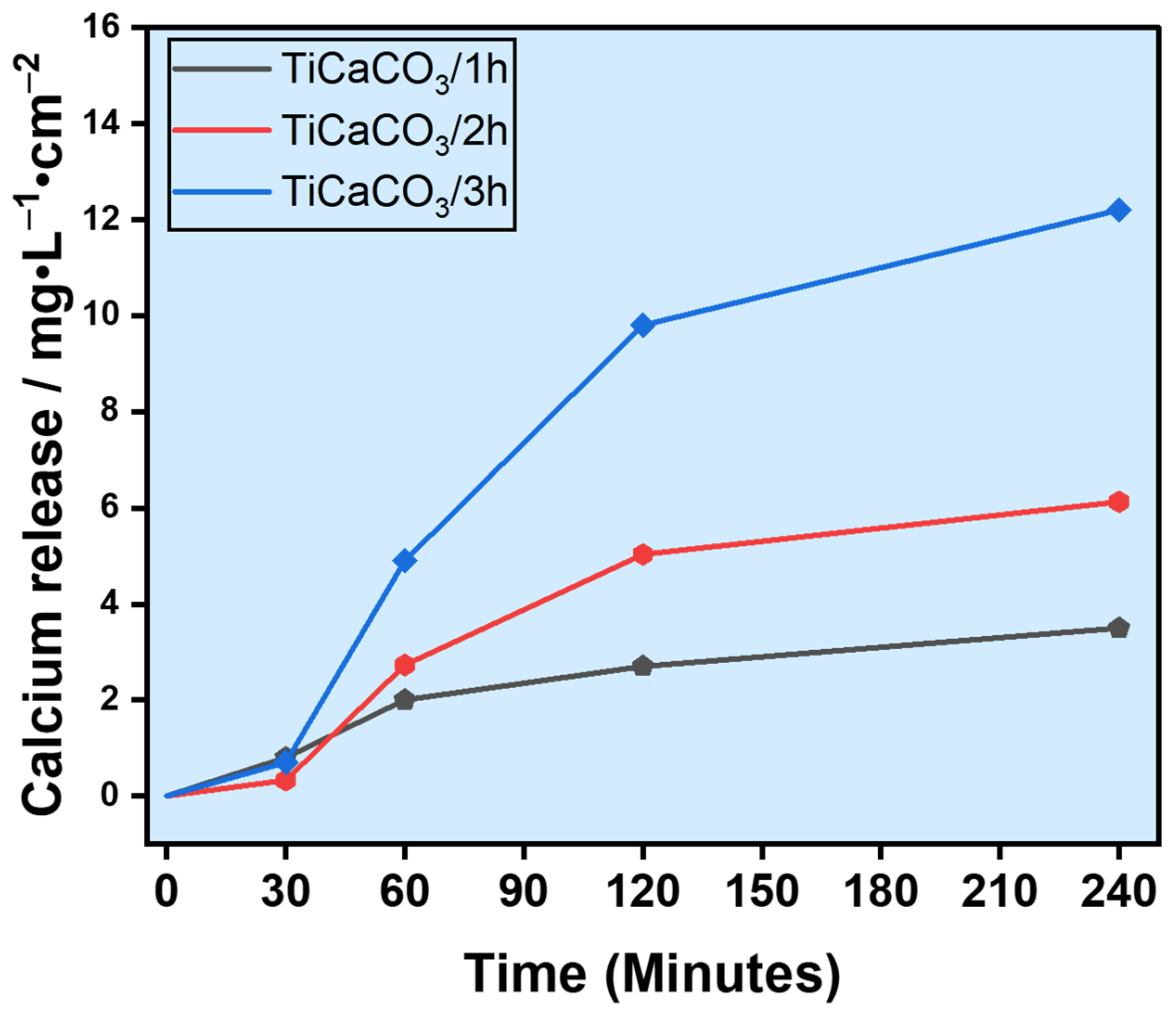
Calcium release profiles of TiCaCO_3_/1h, TiCaCO_3_/2h, and TiCaCO_3_/3h groups.

The calcium release kinetics of deposited layers were assessed by immersion in calcium-free PBS for different time intervals and quantification of ionic species in the solutions via ICP-OES (Figure 3). ICP-OES analysis revealed that all samples exhibited a slower calcium release within the first 30 min, followed by a rapid increase up to 120 min and a sustained release extending to 240 min. The TiCaCO_3_/1h specimens exhibited calcium release of 2.0 mg·L^−1^·cm^−2^ at 60 min, which increased to 2.7 mg·L^−1^·cm^−2^ at 120 min and continued to rise, reaching 3.5 mg·L^−1^·cm^−2^ after 240 min of immersion. Calcium release from the TiCaCO_3_/2h group was 2.73 mg·L^−1^·cm^−2^ at 60 min, progressively increased to 5.03 mg·L^−1^·cm^−2^ at 120 min, and reached 6.13 mg·L^−1^·cm^−2^ after 240 min of incubation. For the TiCaCO_3_/3h group, calcium release was 4.9 mg·L^−1^·cm^−2^ at 60 min, increased to 9.8 mg·L^−1^·cm^−2^ at 120 min, and further rose to 12.2 mg·L^−1^·cm^−2^ after 240 min.

The calcium release kinetics exhibit a clear dependence on coating duration, with longer deposition times producing higher and more sustained ion release. The TiCaCO_3_/1h group provides a controlled, gradual release profile, the TiCaCO_3_/2h group shows an intermediate enhancement, and the TiCaCO_3_/3h group achieves the most pronounced release (Figure 3). This trend underscores coating duration as a critical parameter for tailoring bioactive ion delivery.

**Figure 4:**
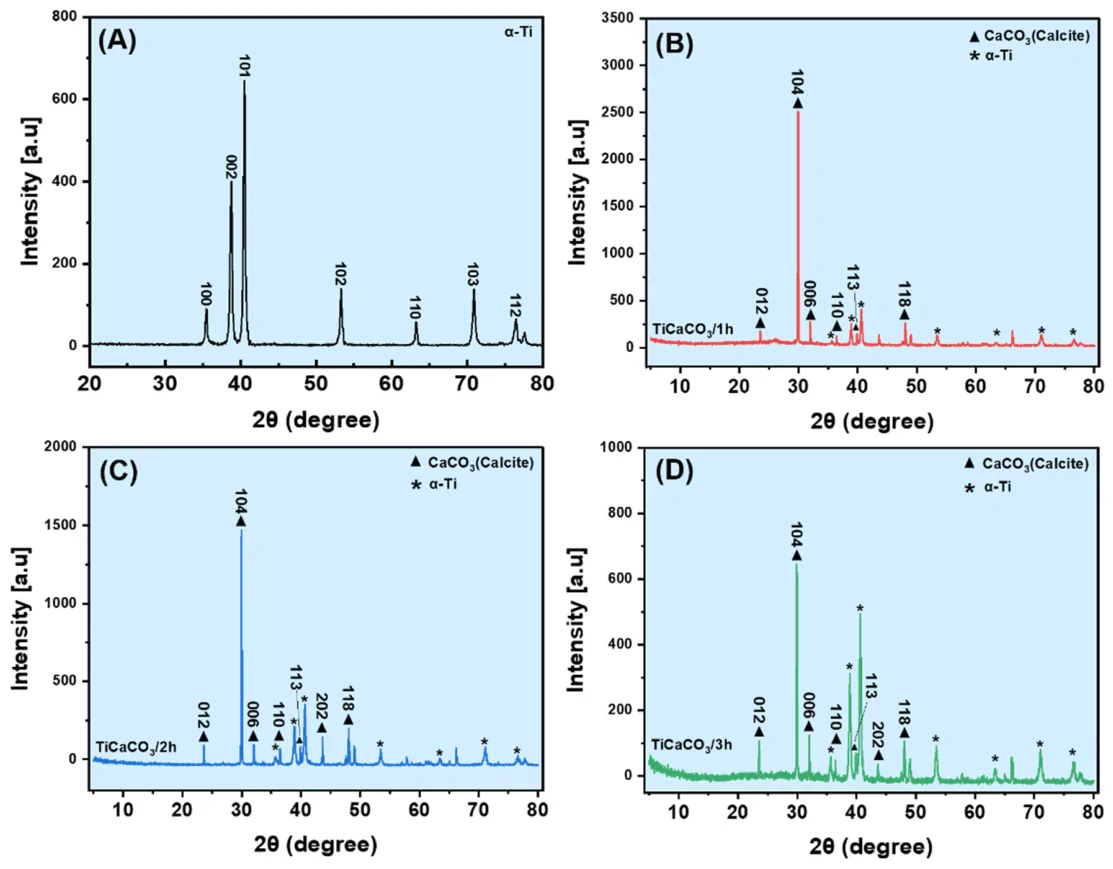
X-ray diffraction patterns of (**A**) Cp Ti, (**B**) TiCaCO_3_/1h, (**C**) TiCaCO_3_/2h, and (**D**) TiCaCO_3_/3h obtained under an applied potential of −1.6 V.

To investigate the crystal structure of the CaCO_3_ scale formed at −1.6 V, XRD analysis was performed, as shown in Figure 4. The X-ray spectrum indicates that the calcium carbonate coating layer on the substrate is composed of calcite crystals, with the dominant crystallographic plane indexed as (104) [32]. The peaks marked with a star (*) are attributed to the Ti underlayer.

The diffraction peaks at 2θ of 35.09°, 38.42°, 40.17°, 53.00°, 62.95°, 70.66°, and 76.22° are assigned to α-Ti crystal planes {100}, {002}, {101}, {102}, {110}, {103}, and {112} in comparison to the powder diffraction card of α-Ti (Figure 4A) (JCPDF 44-1294) [22]. On all the sample groups (TiCaCO_3_/1h, TiCaCO_3_/2h and TiCaCO_3_/3h) the diffraction peaks at 2θ values of 23.4°(012), 29.6°(104), 36.2°(110), 39.6°(113), 43.4°(202), and 47.7°(118), which are consistent with the calcite polymorphic form of CaCO_3_ [33] (Figure 4B–D). These peaks are consistent with data from the Joint Committee on Powder Diffraction Standards (JCPDS No. 72-1214). The dominance of the [26] peak suggests that calcite crystals preferentially nucleate and grow along these planes, which influence the overall morphology and microstructure of the material. The XRD patterns confirm that deposition time does not affect the phase transformation of the crystals at −1.6 V, consistent with the SEM images (Figure 2).

### 3.4. Fibrinogen Adsorption to CaCO_3_ Deposited Titanium

**Figure 5:**
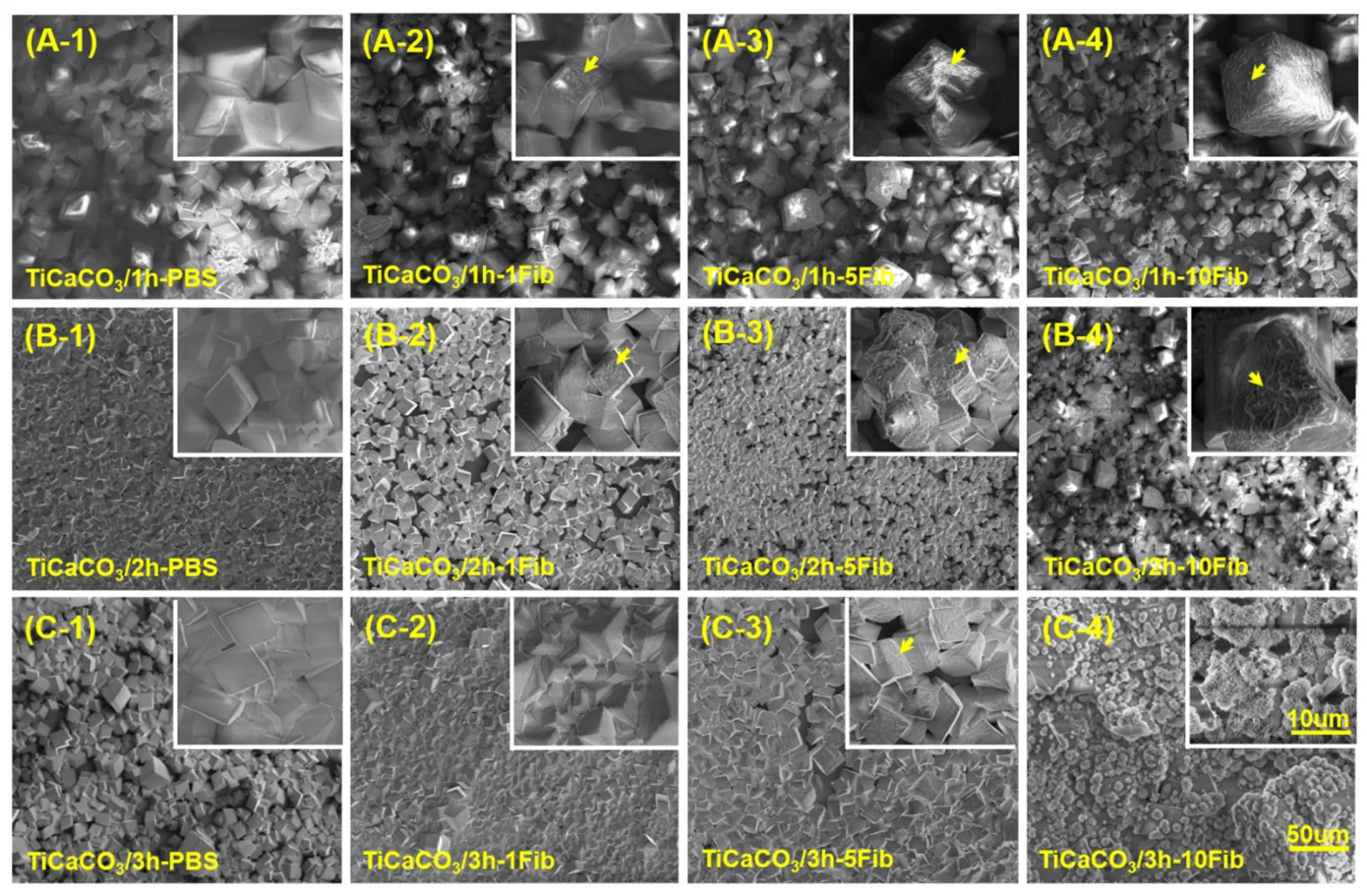
Typical dye-assisted scanning electron microscopy (*d*-SEM) morphologies of the samples after one-hour incubations in PBS (**A-1**,**B-1**,**C-1**), a 1 mg·L^−1^ fibrinogen suspension (**A-2**,**B-2**,**C-2**), a 5 mg·L^−1^ fibrinogen suspension (**A-3**,**B-3**,**C-3**), and a 10 mg·L^−1^ fibrinogen suspension (**A-4**,**B-4**,**C-4**) at 37 °C. The arrows represent the adsorbed fibrinogen layer.

To assess fibrinogen adsorption, the material surfaces were treated with protein-free PBS and fibrinogen suspensions at three concentrations (1 mg·L^−1^, 5 mg·L^−1^, and 10 mg·L^−1^) and incubated for 60 min (Figure 5). Compared to the PBS-incubated counterpart group (Figure 5A-1), no continuous fibrinogen structure was observed on the TiCaCO_3_/1h surface after exposure to the 1 mg·L^−1^, 5 mg·L^−1^, and 10 mg·L^−1^ fibrinogen solutions; only a few short, discontinuous protein fragments were visible (arrows in Figure 5A-2–A-4 inset). Similarly, no integrated protein assembly structure (only some small segments identified, arrowed in the inset of Figure 5B-2–B-4) was detected on the TiCaCO_3_/2h group after incubating in the 1 mg·L^−1^, 5 mg·L^−1^, 10 mg·L^−1^ fibrinogen suspension (Figure 5B-2). Similarly, no continuous fibrillar network was observed on the TiCaCO_3_/3h surface after exposure to the 1 mg·L^−1^ and 5 mg·L^−1^ fibrinogen solution; only a few short, discontinuous protein fragments were visible (Figure 5C-2, arrows in C-3 inset). When the fibrinogen concentration was increased to 10 mg·L^−1^ (Figure 5C-4), a merged, sponge-like fibrinogen fibrin aggregate was formed and was discontinuously distributed across the entire TiCaCO_3_/3h surface after 60 min of incubation. These results show that calcium carbonate deposition for 3 h helps the formation of sponge-like aggregates of fibrinogen on the TiCaCO_3_/3h surface.

To further examine whether PBS interacts with the calcite crystals or induces crystal dissolution, XRD analysis was conducted. All sample groups were incubated for 60 min in PBS or 10 mg·L^−1^ fibrinogen to assess the structural stability of the deposited calcium carbonate coatings under physiological and protein-containing conditions. For TiCaCO_3_/1h, incubation in PBS produced no additional diffraction peaks relative to the unincubated sample, indicating that PBS did not alter the deposited CaCO_3_ crystals or promote detectable phase transformation (Figure S2A, Supplementary Materials). Likewise, incubation with 10 mg·L^−1^ fibrinogen did not generate any new peaks, suggesting that fibrinogen did not induce crystallographic changes in the CaCO_3_ layer. The deposition layer remained predominantly in the calcite phase, with characteristic reflections at 2θ = 29.6. The same trend was observed for TiCaCO_3_/2h **(**Figure S2B, Supplementary Materials) and TiCaCO_3_/3h (Figure S2C, Supplementary Materials), where incubation in either PBS or 10 mg·L^−1^ fibrinogen caused no phase transformation and no appearance of additional calcium carbonate phases. In addition, the persistence of the dominant calcite reflections indicates that neither PBS nor fibrinogen significantly affected the crystal structure or crystallinity of the CaCO_3_ layer. These results indicate that incubation for 60 min with PBS or fibrinogen did not alter the crystallographic structure or crystallinity of the CaCO_3_ layer, demonstrating the good phase stability of the deposited coatings under physiological and protein-containing conditions.

### 3.5. Fibrinogen Adsorption-Induced Binding Energy Shift

**Figure 6:**
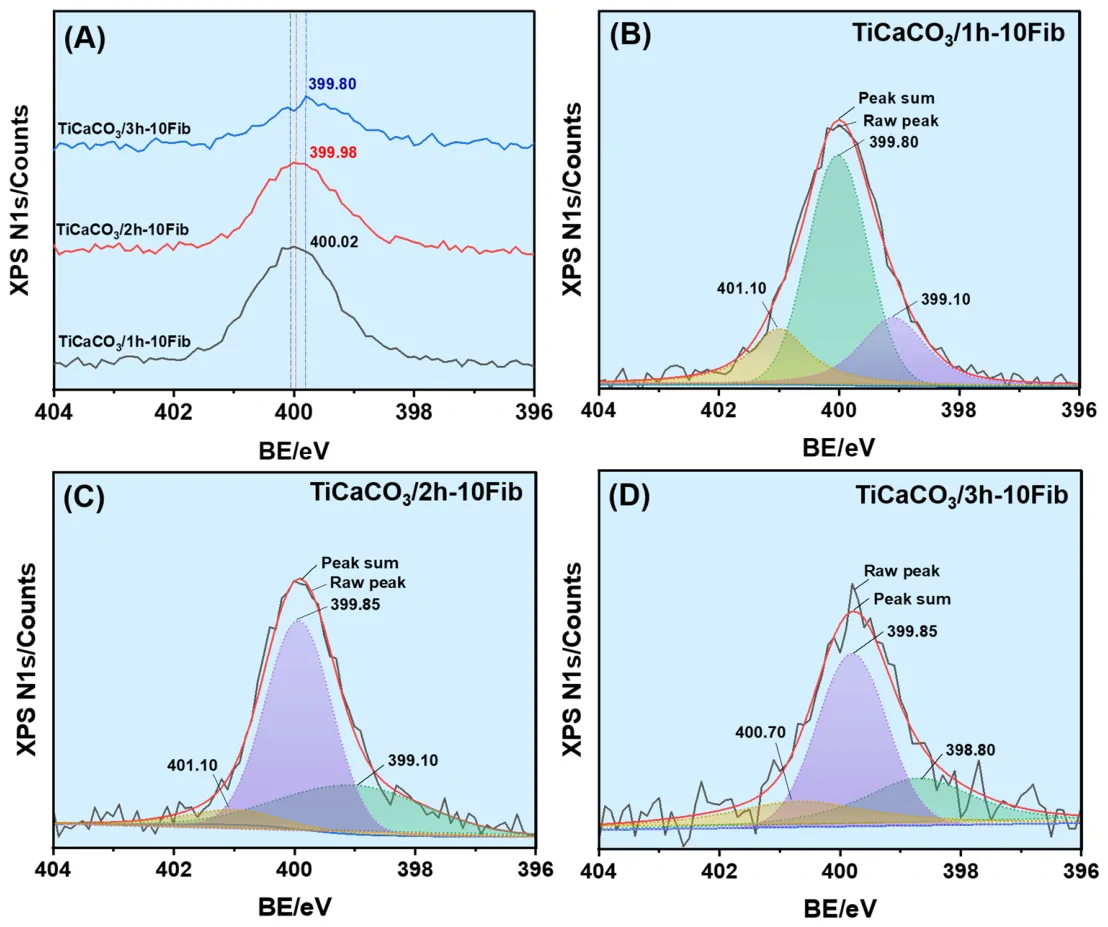
High-resolution XPS N 1s spectra obtained for TiCaCO_3_/1h-10Fib, TiCaCO_3_/2h-10Fib, and TiCaCO_3_/3h-10Fib samples following incubation in a 10 mg·L^−1^ protein solution for 60 min (denoted 10Fib): (**A**) N 1s peak shifts; (**B**) Deconvoluted N 1s spectrum of TiCaCO_3_/1h-10Fib; (**C**) Deconvoluted N 1s spectrum of TiCaCO_3_/2h-10Fib; (**D**) Deconvoluted N 1s spectrum of TiCaCO_3_/3h-10Fib.

To investigate surface–fibrinogen interactions, XPS analysis was conducted after incubating samples in PBS (fibrinogen-free, -PBS) or in a 10 mg·L^−1^ (-10Fib) fibrinogen solution for 60 min. Compared with the PBS controls, the TiCaCO_3_/1h-10Fib, TiCaCO_3_/2h-10Fib, and TiCaCO_3_/3h-10Fib groups exhibited an increased N 1s peak intensity (Figure S3A, Supplementary Materials), confirming fibrinogen adsorption on the material surfaces. The pronounced N 1s signal observed across all functionalized samples indicates that surface nitrogen serves as a reliable quantitative indicator of protein adsorption [34]. XPS analysis further revealed elevated surface nitrogen concentrations for the TiCaCO_3_/1h, TiCaCO_3_/2h, and TiCaCO_3_/3h groups, reaching 4.42 at.%, 3.85 at.%, and 2.17 at.%, respectively, compared with the PBS control (3.15 at.%, 1.91 at.%, and 1.91 at.%, respectively) (Table S1, Supplementary Materials). High-resolution XPS N 1s spectra (Figure 6A) showed peak positions at 400.02 eV, 399.98 eV, and 399.80 eV for TiCaCO_3_/1h-10Fib, TiCaCO_3_/2h-10Fib, and TiCaCO_3_/3h-10Fib, respectively, corresponding to shifts to lower binding energy of 0.04 eV and 0.22 eV for the 2 h and 3 h samples relative to TiCaCO_3_/1h-10Fib. The observed shift in the N 1s binding energy indicates conformational changes in adsorbed fibrinogen across the three groups.

Deconvolution of XPS high-resolution spectra obtained for TiCaCO_3_/1h-PBS and TiCaCO_3_/2h-PBS groups (Figure S3B,C, Supplementary Materials) revealed two peaks at 401.10 eV and 399.80 eV. Deconvolution of TiCaCO_3_/3h-PBS (Figure S3D, Supplementary Materials) revealed one component at 399.80 eV. These binding energy components for all the groups can be assigned to the atmospheric adsorbed nitrogen [35,36]. Deconvolution of the XPS N 1s spectra for the TiCaCO_3_/1h-10Fib and TiCaCO_3_/2h-10Fib groups revealed three components at 401.10 eV, 399.12 eV, and 399.80 eV (Figure 6B,C). The first two components are attributed to protonated and deprotonated amino groups of fibrinogen [37], while the third component is assigned to adsorbed atmospheric nitrogen [35,36,38]. Two additional components at 400.7 eV and 398.8 eV were resolved in the N 1s spectrum of TiCaCO_3_/3h-10Fib (Figure 6D), which are attributed to pyrrole and pyridine nitrogen associated with histidine residues in the fibrinogen structure [38].

**Figure 7:**
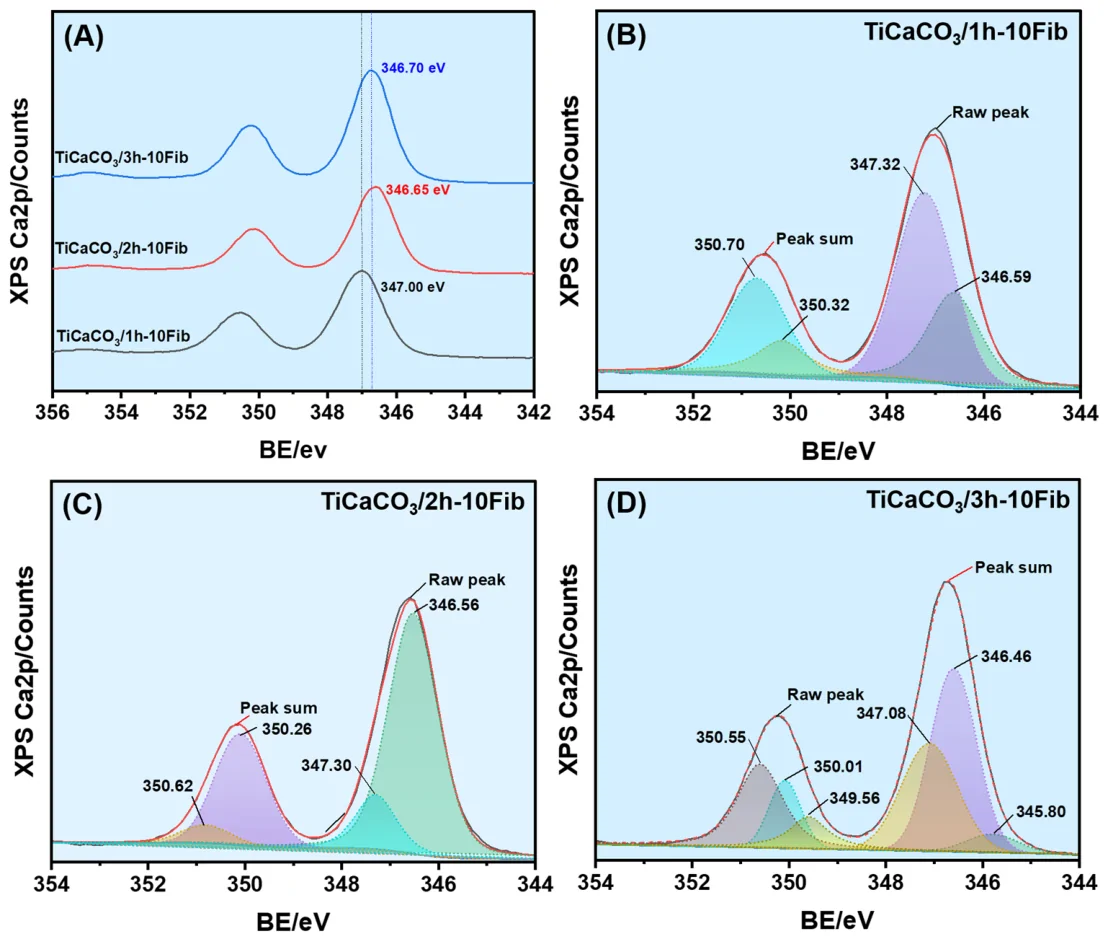
High-resolution XPS Ca 2p spectra obtained for TiCaCO_3_/1h-10Fib, TiCaCO_3_/2h-10Fib, and TiCaCO_3_/3h-10Fib samples following incubation in a 10 mg·L^−1^ protein suspension for 60 min (denoted -10Fib): (**A**) Ca 2p peak shifts; (**B**) Deconvoluted Ca 2p spectrum of TiCaCO_3_/1h-10Fib; (**C**) Deconvoluted Ca 2p spectrum of TiCaCO_3_/2h-10Fib; (**D**) Deconvoluted Ca 2p spectrum of TiCaCO_3_/3h-10Fib.

A similar trend of binding energy shift toward lower values was observed in the high-resolution Ca 2p spectra of TiCaCO_3_/1h-10Fib, TiCaCO_3_/2h-10Fib, and TiCaCO_3_/3h-10Fib following incubation in a 10 mg·L^−1^ protein solution for 60 min, consistent with Ca^2+^ coordination to histidine residues in fibrinogen. The high-resolution Ca 2p peaks were located at approximately 347.00 eV, 346.65 eV, and 346.70 eV, respectively (Figure 7A). Deconvolution of Ca 2p spectra of TiCaCO_3_/1h-PBS, TiCaCO_3_/2h-PBS, and TiCaCO_3_/3h-PBS (Figure S4A–C, Supplementary Materials) identified two doublets. The first two ones at 346.41–346.51 eV (2p3/2)/350.11–350.20 eV (2p1/2) and 347.12–347.21 eV (2p3/2)/350.50–350.85 eV (2p1/2) are attributable to ionic calcium [11,36]. Deconvolution of the Ca 2p spectra for TiCaCO_3_/1h-10Fib (Figure 7B) revealed two doublets at 346.59/350.32 eV and 347.32/350.70 eV.

Similarly, the TiCaCO_3_/2h-10Fib spectrum (Figure 7C) exhibited doublets at 346.56/350.26 eV and 347.30/350.62 eV, characteristic of ionic calcium and shifted to higher binding energies relative to their respective controls (Figure S3A,B). The modest rise in high-binding-energy ionic Ca 2p components between TiCaCO_3_/1h-10Fib and TiCaCO_3_/2h-10Fib (346.56–347.32 eV), relative to their respective PBS controls, reflects weak, non-specific electrostatic Ca carboxylate coordination, which is consistent with the compact, fragmented fibrinogen morphology observed by SEM at these shorter deposition times and limits the protein unfolding required for fibrin assembly. In contrast, deconvolution of the Ca 2p spectrum for TiCaCO_3_/3h-10Fib (Figure 7D) revealed three doublets. The first two, at 347.08/350.55 eV and 346.46/350.26 eV, are attributed to ionic calcium [11], and the third doublet at 345.80/349.56 eV corresponds to neutral calcium [39], suggesting the formation of a zwitterionic complex with histidine residues of fibrinogen [36]. The doublet for TiCaCO_3_/3h-10Fib at 345.80/349.56 eV is markedly lower in binding energy than any ionic component identified at 1 h or 2 h; this lower binding energy indicates increased electron density at the Ca center, consistent with charge donation from histidine imidazole nitrogen and carboxylate oxygen into a neutral, chelation-type Ca protein complex rather than a simple ionic interaction [36].

**Figure 8:**
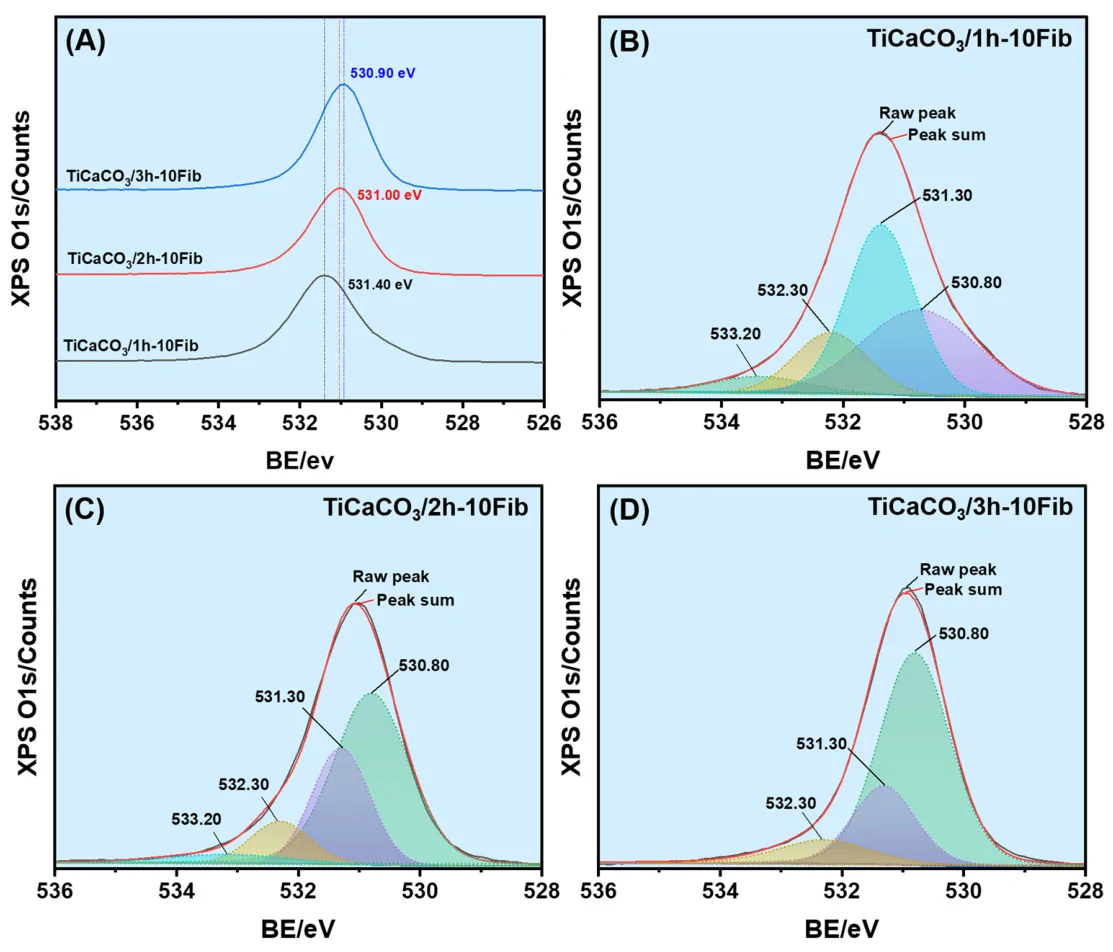
High-resolution XPS O 1s spectra obtained for TiCaCO_3_/1h-10Fib, TiCaCO_3_/2h-10Fib, and TiCaCO_3_/3h-10Fib samples following incubation in a 10 mg·L^−1^ protein suspension for 60 min (denoted -10Fib): (**A**) O 1s peak shifts; (**B**) Deconvoluted O 1s spectrum of TiCaCO_3_/1h-10Fib; (**C**) Deconvoluted O 1s spectrum of TiCaCO_3_/2h-10Fib; (**D**) Deconvoluted O 1s spectrum of TiCaCO_3_/3h-10Fib.

High-resolution XPS O 1s spectra (Figure 8A) showed binding energies of 531.40 eV, 531.00 eV, and 530.90 eV for TiCaCO_3_/1h-10Fib, TiCaCO_3_/2h-10Fib, and TiCaCO_3_/3h-10Fib, respectively. Relative to TiCaCO_3_/1h-10Fib, the 2 h and 3 h samples exhibited downward shifts of 0.40 eV and 0.50 eV, respectively, consistent with the binding energy shift in N 1s as well as Ca2p high-resolution XPS spectra (Figure 6A and Figure 7A). These data suggest that the released calcium can interact simultaneously with nitrogen- and oxygen-containing functional groups in fibrinogen, thereby altering its conformation. Deconvolution of the XPS O 1s spectra of TiCaCO_3_/1h-10Fib and TiCaCO_3_/2h-10Fib (Figure 8B,C) revealed four components. The peak at 533.2 eV was assigned to adsorbed surface water, the peak at 530.8 eV to lattice oxygen [40], the peak at 532.3 eV to Ca–O species [41], and the peak at 531.3 eV to COO^−^ groups in fibrinogen [37]. In contrast, the O 1s spectrum of TiCaCO_3_/3h-10Fib (Figure 8D) exhibited three components at 530.80 eV, 531.30 eV, and 532.30 eV, corresponding to lattice oxygen, COO^−^ groups in histidine, and Ca–O species, respectively [36].

### 3.6. The Amide Band Shifts

**Figure 9:**
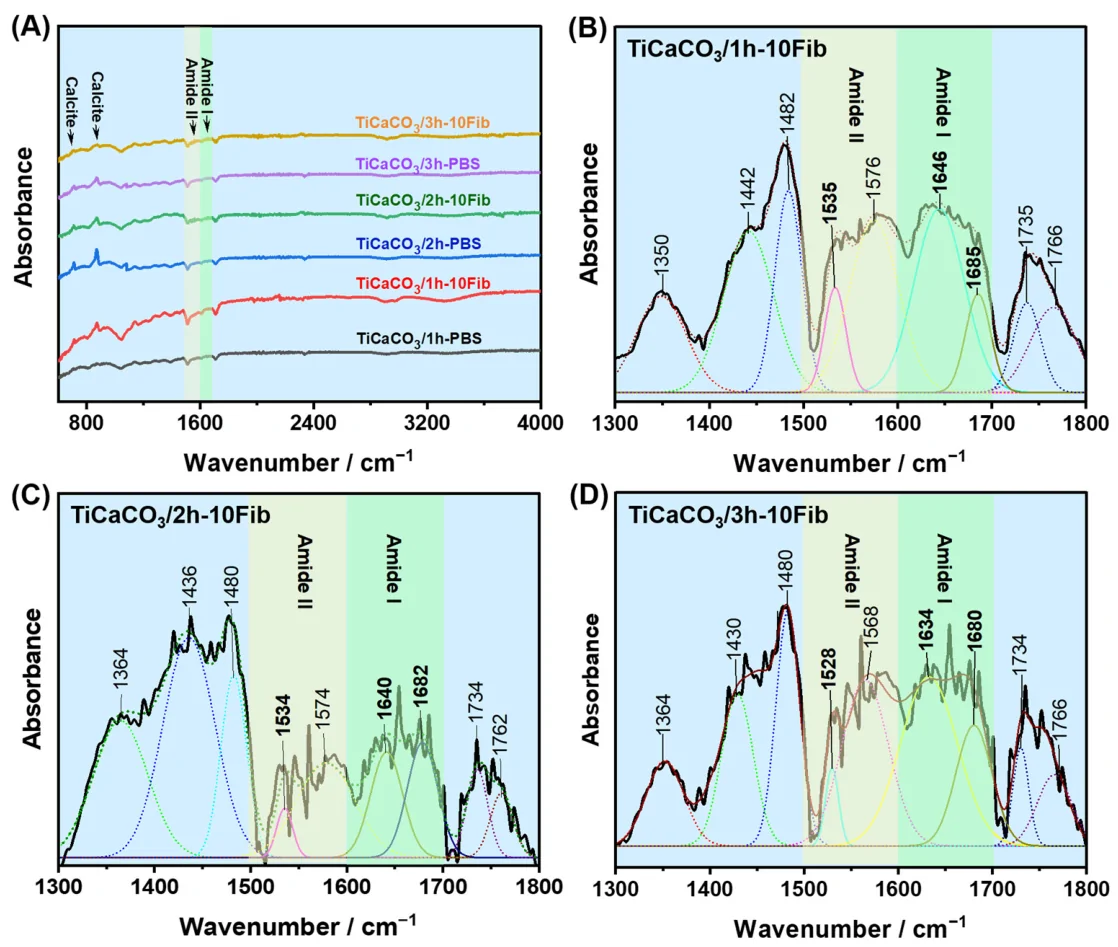
Conformational changes in the adsorbed fibrinogen: (**A**) Fourier-transform infrared spectroscopy (FTIR) spectrum acquired from the TiCaCO_3_/1h, TiCaCO_3_/2h, and TiCaCO_3_/3h after incubating at 37 °C for 1 h in PBS (-PBS) or a suspension of 10 mg·L^−1^ fibrinogen (-10Fib), (**B**) Deconvolution of the representative amide region of the FTIR spectrum acquired on TiCaCO_3_/1h-10Fib, (**C**) TiCaCO_3_/2h-10Fib and (**D**) TiCaCO_3_/3h-10Fib.

The electrochemically deposited CaCO_3_ on Ti groups was further examined by Fourier-transform infrared (FTIR) spectroscopy after incubation with PBS (without fibrinogen) or 10 mg·L^−1^ suspension for 60 min. As shown in Figure 9A, strong Amide I (~1650 cm^−1^), Amide II (~1550 cm^−1^), and Amide B (~3000 cm^−1^) were exclusively detected in all the 3 sample groups after incubation with 10 mg·L^−1^ fibrinogen concentration for 60 min. Also characteristic IR absorbance bands at 876, 848, and 714 cm^−1^ confirm the presence of calcite as the dominant calcium carbonate polymorph [42], consistent with SEM analysis (Figure 2) and XRD spectra of the respective groups (Figure 4). The relative intensity of these bands in sample TiCaCO3/1h-PBS after 1 h deposition remains low, reflecting limited calcite formation. With increasing deposition time from 1 h to 3 h (TiCaCO_3_/2h-PBS and TiCaCO_3_/3h-PBS, respectively), the relative intensity of these characteristic calcite bands progressively increases, indicating enhanced calcite deposition and coating development (Figure 9A). The FTIR spectra revealed a band in the 2372~2313 cm^−1^ range across all TiCaCO_3_ samples with adsorbed fibrinogen (TiCaCO_3_/1h-10Fib: 2372 cm^−1^; TiCaCO_3_/2h-10Fib: 2315 cm^−1^; TiCaCO_3_/3h-10Fib: 2313 cm^−1^). This absorption feature corresponds to the asymmetric stretching vibration of atmospheric CO_2_, a common artifact observed in FTIR measurements when samples are exposed to ambient air during spectral acquisition. The slight wavenumber shifts and intensity variations across deposition times likely reflect differences in surface adsorption of CO_2_ molecules onto the CaCO_3_-coated surfaces, facilitated by the basic nature of calcite [43].

Deconvolution of the Amide I and Amide II bands in the FTIR spectrum acquired from TiCaCO_3_/1h-10Fib (Figure 9B) revealed sharp bands at 1685 cm^−1^, 1646 cm^−1^, 1576 cm^−1^, and 1535 cm^−1^ which were well-identified. The former band originates from the C=O stretching coupled with the in-phase N-H bending and C-N stretching in the β-sheet structures of the adsorbed fibrinogen. The band around 1685 cm^−1^ is assigned to β-turn structures, while the characteristic band for random coil conformation is located at 1646 ± 2 cm^−1^ [44]. The bands in the Amide II region at 1576 cm^−1^ are mainly from asymmetric stretching of the COO^−^ group of adsorbed fibrinogens, while the band around 1535 cm^−1^ can be assigned to symmetric deformation of the N-H group, along with a band around 1482 cm^−1^ of adsorbed fibrinogen. Thus, these bands arise due to the adsorption of aspartic acid residues of fibrinogen, which is consistent with the SEM of the TiCaCO_3_/1h-10Fib group, as no fibrinogen aggregate structure is observed on it [45]. Significant calcium-associated shifts in the amide I and amide II bands were observed for adsorbed fibrinogen across deposition times. The amide I peak for the TiCaCO_3_/3h-10Fib sample shifted to 1634 cm^−1^, representing a 12 cm^−1^ red shift compared to TiCaCO_3_/1h-10Fib (1646 cm^−1^). Similarly, the amide II peak of TiCaCO_3_/3h-10Fib shifted to 1568 cm^−1^), exhibiting an 8 cm^−1^) shift relative to TiCaCO_3_/1h-10Fib.

Since amide II primarily arises from in-plane N-H bending and C-N stretching vibrations [11,45], these shifts suggest calcium-induced deprotonation of N-H groups, potentially involving histidine residues, and formation of calcium-protein complexes due to Ca^2+^ release from the thicker calcite coating (Figure 3). This deprotonation is expected to facilitate the formation of calcium-protein complexes, driven by Ca^2+^ release. These findings suggest that calcium ions initially interact with the fibrinogen’s carboxyl groups and subsequently coordinate with nitrogen-containing groups, such as those in histidine, thereby stabilizing the protein surface interaction. Overall, this calcium-mediated binding mechanism provides a coherent explanation for the observed spectral shifts and highlights the role of coating thickness in modulating fibrinogen adsorption behavior and interfacial chemistry.

## 4. Discussion

Fibrinogen adsorption onto biomaterial surfaces plays a pivotal role in directing the earliest cellular and biological reactions at the material–tissue boundary [46]. Once a biomaterial is implanted, its surface is immediately exposed to physiological fluids, where proteins rapidly adsorb and form the initial biointerface. This adsorbed protein layer ultimately influences biocompatibility, inflammatory responses, thrombosis, and overall clinical performance. Recent studies on CaCO_3_ nanoparticles and coatings demonstrated their potential for drug delivery [18], enhanced osteoblast responses [19], protection against key pathogens without cytotoxicity [20], and superior hemostatic activity [21]. These findings position CaCO_3_ not merely as a bioactive coating but as a multifunctional platform whose protein interactions and biological signaling can be engineered through polymorph control and deposition methods. Building on this concept, the present work examines how electrochemically deposited CaCO_3_ on Ti modulates fibrinogen adsorption and assembly, with the broader goal of establishing controllable fibrinogen-based surface modification. The findings indicate that CaCO_3_ deposition on titanium at −1.6 V for 3 h appears to promote sponge-like fibrin aggregates through calcium-ion-driven conformational changes in adsorbed fibrinogen. Ca^2+^ released from the thicker calcite layer interacts with histidine- and carboxylate-containing regions, specifically the GHRP motif (β15–β18), thereby facilitating intermolecular and interamolecular association and porous, sponge-like fibrillar formation.

**Figure 10:**
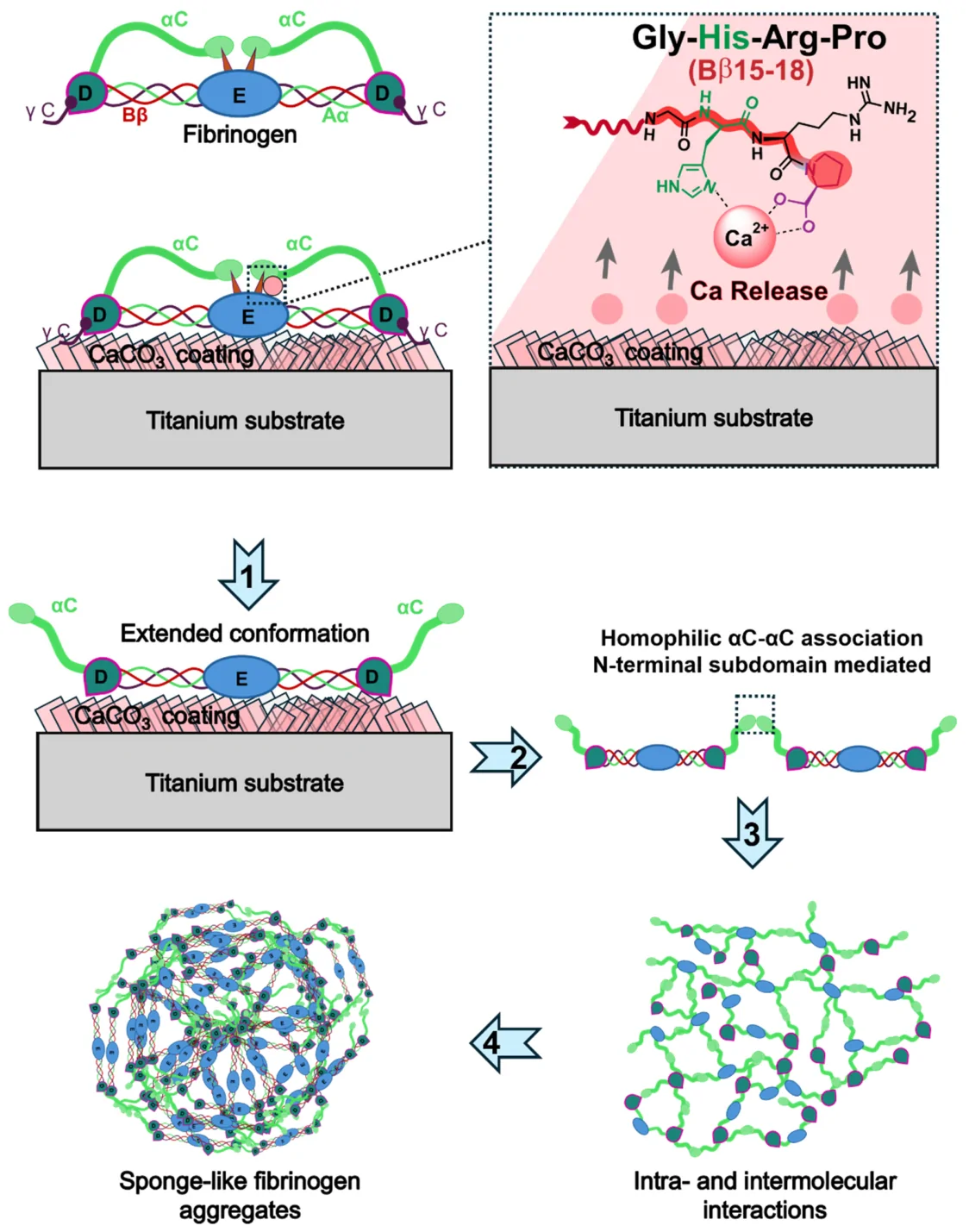
Proposed mechanism of Ca^2+^ coordination with histidine and carboxylate residues in the fibrinogen Bβ-chain segment Gly–His–Arg–Pro (β15–β18) and subsequent fibrinogen aggregate formation on calcite-phase CaCO_3_ thick-coated Ti substrates.

Fibrinogen is a dimeric glycoprotein composed of Aα, Bβ, and γ chains, with the C-terminal regions forming the D domains and the N-terminal regions linked by disulfide bonds to the central E domain. Fibrin formation is initiated by thrombin-mediated cleavage of fibrinopeptides A and B, accompanied by dissociation of the αC domains. The resulting fibrinogen monomers self-assemble into protofibrils through D:E interactions, D:D lateral associations, and αC-αC interactions, which collectively drive higher-order network formation [47]. On a polished Ti substrate, coatings with calcite crystals have been deposited at −1.6 V with their thickness increasing as a function of applied time, as evidenced by SEM analysis (Figure 2) and XRD spectra (Figure 4). On TiCaCO_3_/1h, only short, discontinuous protein fragments were detected even at low fibrinogen concentrations, and increasing to 10 mg·L^−1^ primarily promoted denatured fibrinogen fragments rather than organized protein assembly as evident by *d-*SEM (Figure 5A-1–A-4). Similarly, the TiCaCO_3_/2h surface showed fragmented adsorption at lower concentrations as well as 10 mg·L^−1^ (Figure 5B-1–B-4). In contrast, the TiCaCO_3_/3h coating demonstrated progressive fibrinogen aggregate formation: discontinuous fragments at 1~5 mg·L^−1^, and a sponge-like aggregate of fibrin at 10 mg·L^−1^ (Figure 5C-1–C-4). The formation of sponge-like fibrinogen aggregates is initiated by the release of calcium ions from the material, which specifically bind to the segment Gly–His–Arg–Pro (β15–β18) within fibrinogen, inducing a conformational transition that exposes the cryptic αC-domains. ICP-OES confirmed that TiCaCO_3_/3h exhibited the highest calcium release into the local environment (Figure 3), and deconvolution of the XPS N 1s (Figure 6A,D), Ca2p (Figure 7A,D), and O1s (Figure 8A,D) spectra for TiCaCO_3_/3h evidences energy shifts and components consistent with mixed ionic/netural Ca species and chelation-like environments, revealing the coordination of Ca with histidine (N 1s binding energy component at 398.80 eV) and carboxylate residue in fibrinogen [11,36]. Furthermore, the CaCO_3_ coating thickness-dependent shift in deconvoluted Amide I and Amide II bands (Figure 9B–D) also provides evidence of calcium chelation with histidine. As shown in Figure 10, the gradual exposure of fibrinogen to the calcite-coated Ti surface likely induces an extended αC-domain conformation, which facilitates the first intermolecular recognition events (arrow 1, Figure 10). This structural relaxation promotes homophilic association between the N-terminal subdomains of adjacent αC-domains, most likely through a domain-swapping-like mechanism, thereby initiating oligomer formation (arrow 2, Figure 10). These early αC-αC oligomers function as molecular bridges that draw neighboring fibrinogen assemblies into closer proximity and provide a scaffold for further assembly development [48].

In the next stage, additional intra- and intermolecular contacts involving the C-terminal subdomains and flexible C-connectors reinforce the assembly, consolidating the growing fibrinogen structure into a more cohesive and mechanically stable framework (arrow 3, Figure 10). With continued αC-mediated bridging and cross-linking, these local interactions propagate into a higher-order three-dimensional organization [48]. Thus, yielding a dense yet porous sponge-like fibrinogen matrix in which multiple fibers are interconnected through αC polymers (arrow 4, Figure 10). Together, this hierarchical sequence transforms initially adsorbed fibrinogen into an integrated aggregate with enhanced structural integrity, flexibility, and porosity. Overall, these findings suggest that the formation of sponge-like fibrinogen aggregates is not merely a morphological event but may have biological relevance at the blood–material interface. Fibrinogen, as a major plasma glycoprotein involved in hemostasis, platelet adhesion, inflammation, and wound healing, can strongly influence subsequent protein-cell interactions. The interconnected porous architecture observed here may therefore provide a more stable and functional protein network, potentially favoring interfacial retention, structural integrity, and the early biological responses that regulate thrombotic and healing processes on implant surfaces. Such investigations could be of outstanding interest in hemostasis, particularly in relation to thrombosis-related diseases.

## 5. Conclusions

This study demonstrates that electrochemical deposition of CaCO_3_ on titanium significantly modulates fibrinogen adsorption in a time-dependent manner. Among the tested coatings, the 3h-deposited calcite layer most effectively promoted fibrinogen organization into sponge-like aggregates, indicating that the surface chemistry and calcium availability strongly influence protein conformation necessary for fibrinogen aggregate formation. Mechanistically, the Ca^2+^ release from the calcite coating induces conformational rearrangement of fibrinogen through coordination interactions with specific amino acid residues. In particular, Ca^2+^ forms zwitterionic coordination or chelation complexes with carboxylate groups and nitrogen-containing residues such as histidine within the Bβ chain segment (Gly–His–Arg–Pro, β15–β18), driving the observed macro-assembly into sponge-like aggregates. From a biomaterials perspective, these findings suggest that tailoring CaCO_3_ coating thickness and calcium ion availability is a viable strategy to direct protein conformation and interfacial bioactivity. The resulting fibrinogen architectures may enhance osteoblast adhesion and support favorable osseointegration while potentially mitigating adverse thrombogenic or inflammatory responses. Overall, this work offers a mechanistic framework for designing calcium-modified titanium surfaces that actively regulate protein surface interactions, paving the way for improved performance of next-generation implantable materials.

## Data Availability

The original contributions presented in the study are included in the article, further inquiries can be directed to the corresponding author.

## Supplementary Materials

The following supporting information can be downloaded at: https://www.mdpi.com/article/10.3390/jfb17080366/s1, Figure S1: Structural characteristics of CaCO_3_ crystals: (A) crystal size; (B) surface coverage (%) The CaCO_3_ crystals exhibit a deposition-time-dependent increase in crystal size and surface coverage. With longer deposition times, the crystals become larger and more compact, while the exposed Ti regions progressively disappear, indicating the formation of a more continuous and denser CaCO_3_ layer. Figure S2. X-ray diffraction patterns of (A) TiCaCO_3_/1h, TiCaCO_3_/1h-PBS: 60 min incubation in PBS, TiCaCO_3_/1h-10Fib: 60 min incubation with 10 mg·L^−1^ of fibrinogen (B) TiCaCO_3_/2h, TiCaCO_3_/2h-PBS: 60 min incubation in PBS, TiCaCO_3_/2h-10Fib: 60 min incubation with 10 mg·L^−1^ of fibrinogen (C) TiCaCO_3_/3h, TiCaCO_3_/3h-PBS: 60min incubation in PBS, TiCaCO_3_/3h-10Fib: 60 min incubation with 10 mg·L^−1^ of fibrinogen. XRD analysis demonstrates that the deposited CaCO_3_ coatings remain structurally stable after 60 min incubation in either PBS or fibrinogen solution. For TiCaCO_3_/1h, 2h, and 3h samples, no additional diffraction peaks were detected relative to the unincubated controls, indicating that neither PBS nor fibrinogen induced phase transformation or dissolution of the calcite crystals. The dominant reflections at 2θ ≈ 29.6° corresponding to calcite persisted across all groups, while α Ti peaks remained unchanged, confirming substrate stability. Figure S3. XPS N 1s spectra acquired from TiCaCO_3_/1h, TiCaCO3/2h and TiCaCO_3_/3h groups after 60 min incubations in PBS (without fibrinogen, denoted -PBS) or 10 mg·L^−1^ protein suspension (denoted -10Fib) for 60 min: (A) Survey spectra; (B) Deconvolution of the N 1s spectrum acquired from TiCaCO_3_/1h-PBS; (C) Deconvolution of the N 1s spectrum acquired from TiCaCO_3_/2h-PBS; (D) Deconvolution of the N 1s spectrum acquired from TiCaCO_3_/3h -PBS. XPS analysis confirms fibrinogen adsorption on TiCaCO_3_ coatings. Compared with PBS controls, all fibrinogen incubated groups (TiCaCO_3_/1h 10Fib, TiCaCO_3_/2h 10Fib, TiCaCO_3_/3h 10Fib) exhibited increased N 1s peak intensity, consistent with protein deposition on the surfaces. Deconvolution of the high resolution N 1s spectra for TiCaCO_3_/1h PBS and TiCaCO_3_/2h PBS revealed two components at 401.10 eV and 399.80 eV, while TiCaCO_3_/3h PBS showed a single component at 399.80 eV, attributable to atmospheric adsorbed nitrogen. Figure S4. High-resolution XPS Ca 2p spectra obtained for TiCaCO_3_/1h, TiCaCO_3_/2h, and TiCaCO_3_/3h samples following incubation in PBS for 60 min (denoted -PBS): (A) Deconvoluted Ca 2p spectrum of TiCaCO_3_/1h-PBS; (B) Deconvoluted Ca 2p spectrum of TiCaCO_3_/2h-PBS; (C) Deconvoluted Ca 2p spectrum of TiCaCO_3_/3h-PBS. The high resolution XPS Ca 2p spectra of TiCaCO_3_ coatings after PBS immersion consistently exhibit two well defined doublet peaks corresponding to Ca 2p_3_/_2_ (≈350.0–350.8 eV) and Ca 2p_1_/_2_ (≈346.4–347.2 eV). These binding energies are characteristic of ionic Calcium in carbonate environments, confirming the chemical stability of the deposited CaCO_3_ phase. Table S1. X-ray photoelectron spectroscopy elemental composition of samples after 60 min of incubation in calcium-free phosphate-buffered saline (PBS) or in a 10 mg·L^−1^ fibrinogen suspension.
