# Supplementary material for: Fibrinogen Adsorption and Sponge-like Aggregate Formation on Titanium Modified by Electrochemically Deposited CaCO3
Source: J Funct Biomater. 2026 Jul 30;17(8):366. doi: 10.3390/jfb17080366 (PMC13513941; doi:10.3390/jfb17080366)
Supplement: Supplementary file 1 [file jfb-17-00366-s001.zip › jfb-4413965-supplementary.pdf]

Supplementary

Fibrinogen Adsorption and Sponge-like Aggregates Formation on Titanium Modified by Electrochemically Deposited $CaCO_3$

Table S1

Table S1. X-ray photoelectron spectroscopy elemental composition of samples after 60 minutes of incubation in calcium-free phosphate-buffered saline (PBS) or in a 10 mg•L^{-1} fibrinogen suspension.

Samples	Incubations	C (at.%)	N (at.%)	Ti (at.%)	O (at.%)	Ca (at.%)
$TiCaCO_3$/1h	PBS	45.25	3.15	0.67	40.41	10.52
	10mg•L^{-1} Fib	35.32	4.42	2.29	46.28	11.69
$TiCaCO_3$/2h	PBS	34.18	1.91	1.43	50.30	12.17
	10mg•L^{-1} Fib	37.44	3.85	0.65	45.81	12.26
$TiCaCO_3$/3h	PBS	36.09	1.71	0.56	48.40	13.23
	10mg•L^{-1} Fib	31.92	2.17	0.47	50.38	15.07

Figure S1

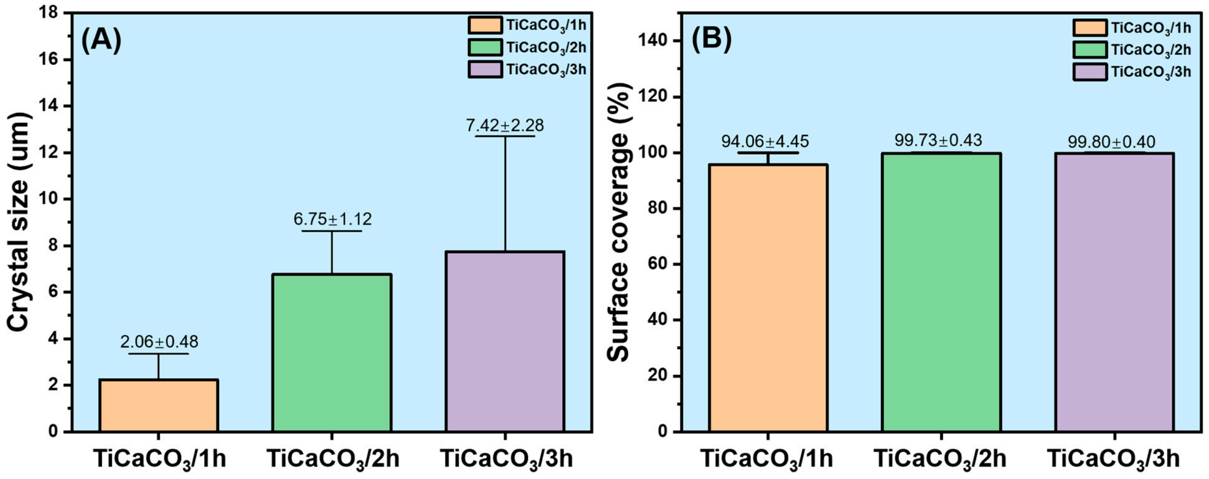

Figure S1. Structural characteristics of $CaCO_3$ crystals: **(A)** crystal size; **(B)** surface coverage (%) The $CaCO_3$ crystals exhibit a deposition-time-dependent increase in crystal size and surface coverage. With longer deposition times, the crystals become larger and more compact, while the exposed Ti regions progressively disappear, indicating the formation of a more continuous and denser $CaCO_3$ layer.

Figure S2

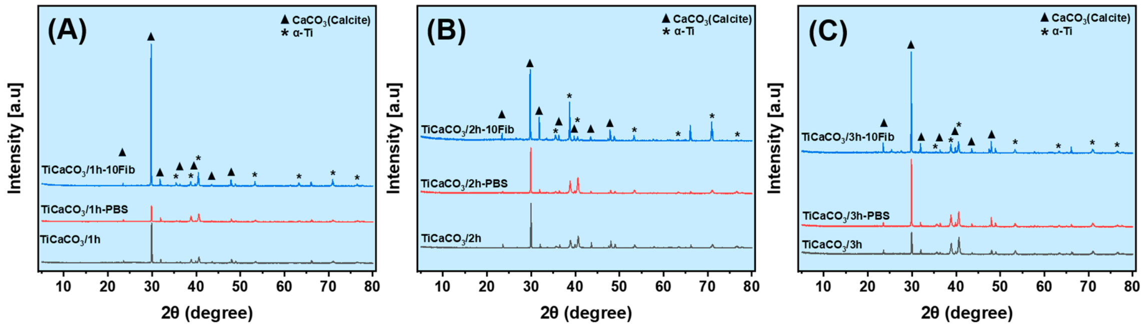

Figure S2. X-ray diffraction patterns of (**A**) $TiCaCO_3$/1h, $TiCaCO_3$/1h-PBS: 60 min incubation in PBS, $TiCaCO_3$/1h-10Fib: 60 min incubation with 10 mg•L^{-1} of fibrinogen (**B**) $TiCaCO_3$/2h, $TiCaCO_3$/2h-PBS: 60 min incubation in PBS, $TiCaCO_3$/2h-10Fib: 60 min incubation with 10 mg•L^{-1} of fibrinogen (**C**) $TiCaCO_3$/3h, $TiCaCO_3$/3h-PBS: 60min incubation in PBS, $TiCaCO_3$/3h-10Fib: 60 min incubation with 10 mg•L^{-1} of fibrinogen. XRD analysis demonstrates that the deposited $CaCO_3$ coatings remain structurally stable after 60 min incubation in either PBS or fibrinogen solution. For $TiCaCO_3$/1h, 2h, and 3h samples, no additional diffraction peaks were detected relative to the unincubated controls, indicating that neither PBS nor fibrinogen induced phase transformation or dissolution of the calcite crystals. The dominant reflections at 2θ ≈ 29.6° corresponding to calcite persisted across all groups, while α-Ti peaks remained unchanged, confirming substrate stability.

Figure S3

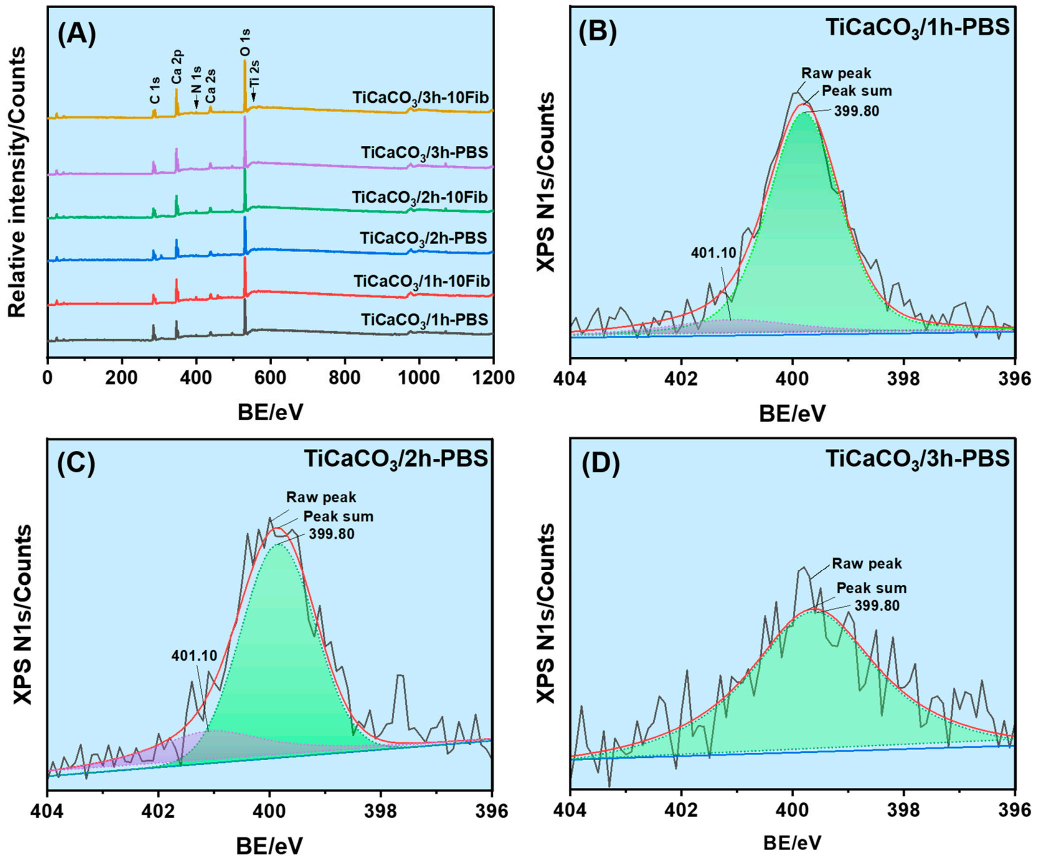

Figure S3. XPS N1s spectra acquired from $TiCaCO_3$/1h, $TiCaCO_3$/2h and $TiCaCO_3$/3h groups after 60 min incubations in PBS (without fibrinogen, denoted -PBS) or 10 mg•L^{-1} protein suspension (denoted -10Fib) for 60 min: (A) Survey spectra; (B) Deconvolution of the N 1s spectrum acquired from $TiCaCO_3$/1h-PBS; (C) Deconvolution of the N 1s spectrum acquired from $TiCaCO_3$/2h-PBS; (D) Deconvolution of the N 1s spectrum acquired from $TiCaCO_3$/3h -PBS. XPS analysis confirms fibrinogen adsorption on $TiCaCO_3$ coatings. Compared with PBS controls, all fibrinogen-incubated groups ($TiCaCO_3$/1h-10Fib, $TiCaCO_3$/2h-10Fib, $TiCaCO_3$/3h-10Fib) exhibited increased N 1s peak intensity, consistent with protein deposition on the surfaces. Deconvolution of the high-resolution N 1s spectra for $TiCaCO_3$/1h-PBS and $TiCaCO_3$/2h-PBS revealed two components at 401.10 eV and 399.80 eV, while $TiCaCO_3$/3h-PBS showed a single component at 399.80 eV, attributable to atmospheric adsorbed nitrogen.

Figure S4

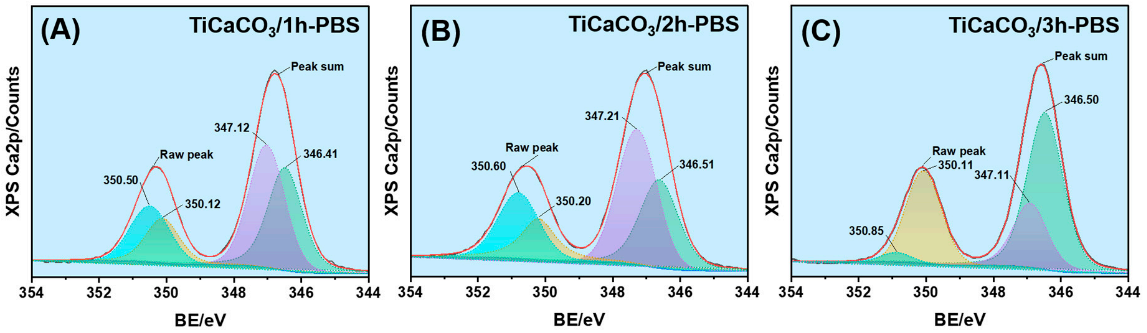

Figure S4. High-resolution XPS Ca 2p spectra obtained for $TiCaCO_3$/1h, $TiCaCO_3$/2h, and $TiCaCO_3$/3h samples following incubation in PBS for 60 min (denoted -PBS): (**A**) Deconvoluted Ca 2p spectrum of $TiCaCO_3$/1h-PBS; **(B)** Deconvoluted Ca 2p spectrum of $TiCaCO_3$/2h-PBS; (**C**) Deconvoluted Ca 2p spectrum of $TiCaCO_3$/3h-PBS. The high-resolution XPS Ca 2p spectra of $TiCaCO_3$ coatings after PBS immersion consistently exhibit two well-defined doublet peaks corresponding to Ca $2p_3/_2$ (≈350.0–350.8 eV) and Ca $2p_1/_2$ (≈346.4–347.2 eV). These binding energies are characteristic of ionic Calcium in carbonate environments, confirming the chemical stability of the deposited $CaCO_3$ phase.
